# Iodinated Copper–Cysteamine Nanoparticles as Radiosensitizers for Tumor Radiotherapy

**DOI:** 10.3390/pharmaceutics17020149

**Published:** 2025-01-22

**Authors:** Miaomiao Zhang, Yu Yang, Ying Xu, Jie Wang, Shihong Li

**Affiliations:** 1 State Key Laboratory of Radiation Medicine and Protection, School of Radiation Medicine and Protection, Suzhou Medical College of Soochow University, Suzhou 215123, China; 20224220061@stu.suda.edu.cn (M.Z.); yangyu6335@126.com (Y.Y.); 20224020015@stu.suda.edu.cn (Y.X.); 2 Department of Clinical Pharmacology, The First Affiliated Hospital of Soochow University, Suzhou 215006, China; 3 Institute for Advanced Materials, School of Material Science and Engineering, Jiangsu University, Zhenjiang 212013, China; wangjie@ujs.edu.cn

**Keywords:** copper–cysteamine nanoparticles, radiosensitization, radiotherapy, X-ray-induced photodynamic therapy, ^131^I

## Abstract

**Background/Objectives:** Radiotherapy is a widely applied first-line clinical treatment modality of cancer. Copper–cysteamine (Cu-Cy) nanoparticles represent a new type of photosensitizer that demonstrates significant anti-tumor potential by X-ray-induced photodynamic therapy. Iodide is a high-Z element with superior X-ray absorption ability and has the β-decay radiotherapeutic nuclide, ^131^I, which emits Cherenkov light. In this study we aimed to investigate the X-ray-induced photodynamic therapy potential of iodinated Cu-Cy (Cu-Cy-I) nanoparticles and also explore the local treatment efficacy of ^131^I-labeled Cu-Cy-I ([^131^I]Cu-Cy-I) nanoparticles. **Methods**: The synthesis of [^131^I]Cu-Cy-I nanoparticles was performed with [^131^I]I^−^ anions. The in vitro radiobiological effects on tumor cells incubated with Cu-Cy-I nanoparticles by X-ray irradiation were investigated. The in vivo tumor growth-inhibitory effects of the combination of Cu-Cy-I nanoparticles with X-ray radiotherapy and [^131^I]Cu-Cy-I nanoparticles were evaluated with 4T1 tumor-xenografted mice. **Results**: The in vitro experiment results indicated that the X-ray irradiation with the presence of Cu-Cy-I nanoparticles produced a higher intracellular reactive oxygen species (ROS) level and more DNA damage of 4T1 cells and showed a stronger tumor cell killing ability compared to X-ray irradiation alone. The in vivo experimental results with 4T1 breast carcinoma-bearing mice showed that the combination of an intratumoral injection of Cu-Cy-I nanoparticles and X-ray radiotherapy enhanced the tumor growth-inhibitory effect and prolonged the mice’s lives. **Conclusions**: Cu-Cy-I nanoparticles have good potential as new radiosensitizers to enhance the efficacy of external X-ray radiotherapy. However, the efficacy of local treatment with [^131^I]Cu-Cy-I nanoparticles at a low ^131^I dose was not verified. The effective synthesis of smaller sizes of nanoparticles is necessary for further investigation of the radiotherapy potential of [^131^I]Cu-Cy-I nanoparticles.

## 1. Introduction

Radiotherapy is one of the main first-line clinical therapy modalities used to treat cancers [1]. More than half of cancer patients need radiotherapy [2,3]. However, the radiation doses of radiotherapy are strictly constrained by the side effects of ionizing radiation on normal tissue in the vicinity of the targeted tumor tissue. Radiosensitizers have been urgently developed to improve the sensitivity of tumors to radiotherapy and reduce the side effects as an adjunctive treatment.

Photodynamic therapy (PDT) is a potential and clinically applicable modality to treat cancer which utilizes specific wavelengths of light to excite photosensitizers to induce reactive oxygen species (ROS) to eliminate tumor cells [4,5,6]. Due to the limited tissue penetration ability of visible light (<2 mm) and near-infrared (NIR) light (∼1 cm), clinical PDT is primarily used to treat tumors that are accessible by endoscopy or near the body’s surface [7,8]. To overcome the drawback of the low tissue penetration of PDT, X-ray-induced photodynamic therapy has been proposed as a new treatment modality for deep tumors, combining the benefits of radiotherapy and PDT [9,10,11,12].

Cherenkov radiation (CR) is a well-known phenomenon in nuclear physics. When a charged particle passes through a dielectric medium with a velocity greater than the phase velocity of light, it can induce the polarization of the molecules in the medium; then, blueish-white light will be emitted upon the relaxation of these molecules. The Cerenkov emission spectrum consists of continuous wavelengths from ultraviolet (UV) to visible light and the light intensity decreases with increasing wavelength, following an inversely proportional relationship to the square of the wavelength. The Cerenkov luminescence dominating in the UV region provides the potency as an excitation source to excite or activate UV-responsive photosensitizers. The Cherenkov radiation of radionuclides may also provide a new in situ excitation light source to activate photosensitizers continuously and thus enhance the capability of PDT to treat deeper tumors [13,14,15]. Combinations of radionuclides with photosensitizers to support both radionuclide therapy as well as continuous radiation-induced PDT have been proposed [12,16]. The Cherenkov light intensities of radionuclides are of low levels, as revealed by theoretical estimation, and highly sensitive photochemistry alterations are required for practical therapeutic applications [17]. The therapeutic applications of various CR-activated photosensitizers with radionuclides, such as ^18^F, ^68^Ga, ^89^Zr, ^64^Cu, and ^131^I, have been investigated by in vitro and preclinical studies, as summarized in a review article [18]. ^131^I-carrying photosensitive nanoplatforms have shown inhibitory effects on tumor growth in animal models in recent studies [9,19]. The elevated level of ROS generated within tumor cells by photosensitizers can induce more serious DNA damage and enhance the sensitization of cancer cells to radiotherapy [20,21].

Chlorinated copper–cysteamine (Cu-Cy-Cl) nanoparticles have emerged in recent years as a new type of radiosensitizer and photosensitizer, exhibiting anti-tumor potential by X-ray-induced photodynamic therapy or under other activation conditions, such as UV light, microwave, and ultrasound, showing broad application prospects [22,23,24,25,26]. Iodinated copper–cysteamine (Cu-Cy-I) nanoparticles showed higher stability, lower dark toxicity, and singlet oxygen (^1^O_2_) generation ability and more effective photodynamic therapy (PDT) effects under UV irradiation than Cu-Cy-Cl nanoparticles [27]. Moreover, iodide is a high-Z element with superior X-ray absorption ability and has the widely used therapeutic radionuclide ^131^I, which emits Cherenkov light accompanying β- decay [28]. In this study, we aimed to investigate the X-ray-induced photodynamic therapy potential of Cu-Cy-I nanoparticles and also explore local radionuclide therapy with ^131^I-labeled Cu-Cy-I ([^131^I]Cu-Cy-I) nanoparticles, hypothesizing these radioactive nanoparticles are potential candidates for combined radionuclide therapy and radionuclide-induced photodynamic therapy.

## 2. Materials and Methods

### 2.1. Materials

Cysteamine hydrochloride (Cy·HCl) was purchased from Sigma-Aldrich (Shanghai) Trading Co., Ltd. (Shanghai, China). Copper acetate monohydrate (CuAc_2_·H_2_O) was obtained from Macklin Biochemical Co., Ltd. (Shanghai, China). Potassium iodide (KI) was sourced from Shanghai Roonee Biochemical Technology Co., Ltd. (Shanghai, China). PEG-4000 was acquired from Shanghai Hushi Reagent Factory Co., Ltd. (Shanghai, China). Polyvinyl alcohol (PVA), Hoechst 33342 stain, and Alexa Fluor 647-conjugated goat anti-mouse IgG were purchased from Shanghai Biyuntian Biotechnology Co., Ltd. (Shanghai, China). [^131^I]Sodium iodide solution was obtained from Sichuan Zhonghe Gaotong Pharmaceutical Co., Ltd. (Sichuan, China). The 2′,7′-dichlorodihydrofluorescein diacetate (DCFH-DA) was sourced from Suzhou UElandy Biotechnology Co., Ltd. (Suzhou, China). Phosphorylated Histone H_2_A_X_ (Ser 139) was acquired from Santa Cruz Biotechnology (Shanghai) Co., Ltd. (Shanghai, China). The CCK-8 test kit was supplied by Hangzhou Fude Biological Technology Co., Ltd. (Hangzhou, China). The anti-fade mounting medium with 4′,6-diamidino-2-phenylindole (DAPI) was obtained from Thermo Fisher Scientific (China) Co., Ltd. (Shanghai, China).

### 2.2. Preparation of Cu-Cy-I Nanoparticles

The synthesis of Cu-Cy-I was conducted according to a previous method with some modifications [27,28]. Briefly, 62.0 mg of CuAc_2_·H_2_O was mixed with 10.0 mL of deionized water in a glass vial and stirred vigorously with a magnetic bar until the salt was completely dissolved; then, 70.4 mg of Cy·HCl was added and the resulting solution was pale/light yellow. Upon the addition of 103.0 mg of KI, the solution turned colorless. Then, 7.8 mg of PEG-4000 and 155.0 mg of PVA were added to the reaction system (molar ratio, CuAc_2_/Cy/KI/PEG4000/PVA = 1:2:2:0.00625:0.01) and the pH value was adjusted to about 7.0 using 1 M NaOH. The reaction mixture in the vial was then protected with nitrogen gas and heated at 120 °C in an oil bath. After 5–10 min of heating, a milky white suspension formed in the vial which emitted bright orange fluorescent light under UV light irradiation. The reaction mixture was further heated for 10 min and then cooled to room temperature. The suspension was centrifuged at 12,000 rpm for 10 min. The precipitate was washed with a water and ethanol mixture (volume ratio, 5:4) three times and dried in vacuo to obtain the Cu-Cy-I product.

### 2.3. Characterization and Optical Properties of Cu-Cy-I Nanoparticles

The synthesized Cu-Cy-I nanoparticles were dried and their X-ray diffraction (XRD) spectrum with a 2 theta ranging from 5° to 70° was measured with a D8 Advance XRD spectrometer (Bruker, Dresden, Germany). The hydrodynamic particle sizes and polydispersity index (PDI) of the Cu-Cy-I nanoparticles dispersed in water were determined with a Zetasizer Nano ZS90 analyzer (Malvern Instruments, Malvern, UK). About 0.1 mg of Cu-Cy-I nanoparticles was dispersed in 1.0 mL deionized water and placed in quartz cuvette. The UV–visible absorption spectrum was measured with a UV–visible–near-infrared spectrophotometer (UV-3600, Shimadzu Corporation, Kyoto, Japan). The fluorescence emission spectrum from 380 nm to 800 nm by excitation light at 365 nm and the excitation spectrum from 250 nm to 450 nm by emission light at 600 nm were measured with a fluorescence spectrometer (FLS980-STM, Edinburgh Instruments Ltd., Livingston, UK).

### 2.4. Preparation of [^131^I]Cu-Cy-I Nanoparticles

The ^131^I-labeled [^131^I]Cu-Cy-I nanoparticles were synthesized in a similar procedure to the synthesis of Cu-Cy-I nanoparticles, except the KI solution was pre-mixed with 2 mCi [^131^I]NaI. The synthesized [^131^I]Cu-Cy-I suspension was purified using an Amicon 100 kDa molecular weight cutoff (MWCO) ultrafiltration tube to remove unreacted [^131^I]I^−^, followed by washing with deionized water three times, and then the nanoparticles were resuspended in saline. The radioactivity of the purified [^131^I]Cu-Cy-I nanoparticles was measured with a CRC-55tR dose calibrator (Capintec, Inc., Florham Park, USA). The radiochemical purity of [^131^I]Cu-Cy-I was measured by radio-thin layer chromatography (radio-TLC) with a Mini-Scan TLC scanner (Eckert & Ziegler Medical, Berlin, Germany). Then, 5 µL of [^131^I]Cu-Cy-I suspension was spotted on an iTLC-SG stripe and developed in 85% MeOH/water or saline. The ^131^I-labeled nanoparticles stayed at the origin (Rf = 0) and free [^131^I]I^−^ moved with the solvent front (Rf = 1.0). The labeling efficiency of the [^131^I]Cu-Cy-I nanoparticles was calculated by dividing the ^131^I radioactivity of the purified nanoparticles by the ^131^I radioactivity added into the initial reaction system.

For radiolabeling stability testing, 50 µL of purified [^131^I]Cu-Cy-I nanoparticles was mixed with either 500 µL of phosphate buffered saline (PBS) or 500 µL of 10% fetal bovine serum (FBS). After different incubation periods (0, 2, 8, 12, 24, and 48 h) at 37 °C, the mixture samples were centrifuged through Amicon filters (100 kDa MWCO). The radioactivity of dissociated free [^131^I]I^−^ in eluate was measured and the radiolabeling stability was assessed by calculating the radioactivity ratio of the dissociated free [^131^I]I^−^ in the eluate to the initial [^131^I]Cu-Cy-I sample.

### 2.5. Tumor Cell Culture

The mouse mammary carcinoma 4T1 cell line was obtained from ATCC. Mouse ovarian cancer ID8 cells and human ovarian cancer OVCAR3 and A2780 cells were purchased from Wuhan Punosai Biotechnology Co., Ltd. (Wuhan, China). The 4T1, ID8, OVCAR3, and A2780 cells were cultured in DMEM medium supplemented with 10% FBS, penicillin (100 U/mL), and gentamicin (100 µg/mL) at 37 °C in a humidified 5% CO_2_ incubator. The cells were subcultured every 2–3 days, depending on growth conditions. Cells in the logarithmic growth phase were used for the experiments.

### 2.6. Detection of ROS in Tumor Cells

The reactive oxygen species (ROS) fluorescent probe DCFH-DA was utilized for the detection of intracellular ROS, following the literature method with minor modification [29]. First, 4 × 10^5^ 4T1 cells were seeded in a confocal dish for confocal microscopic imaging and 1 × 10^6^ 4T1 cells were seeded in a well of 6-well plate for flow cytometric analysis. And allowed to adhere for overnight, the cells were incubated with 10 µg/mL Cu-Cy-I nanoparticles suspended in DMEM medium for 12 h. Then, 10 µM of DCFH-DA solution was added into the well. After 30 min of culture at 37 °C, the cells exposed to 6 Gy of X-ray irradiation (IR) with a RS 2000 X-ray irradiator (Rad Source Technologies, Buford, GA, USA) and were further cultured for 15 min. Subsequently, the culture medium was aspirated and the cells were washed with PBS 3 times, followed by adding DMEM medium containing Hoechst 33342 to stain the cell nuclei. Then, the ROS generation within the 4T1 tumor cells cultured in the dishes was measured with a FV1200 confocal laser scanning microscope (Olympus, Tokyo, Japan). The 4T1 cells cultured in the 6-well plate were digested with 0.25% trypsin–EDTA solution and assayed with a FACSVerse flow cytometer (BD Biosciences, Franklin Lakes, NJ, USA). The fluorescent excitation wavelength of the DCF was setup at 488 nm.

### 2.7. γH_2_AX Immunofluorescence of Tumor Cells

For the DNA damage assay, 5 × 10^4^ 4T1 cells were seeded in a well of a 24-well plate with a glass slide at the bottom and cultured with DMEM medium at 37 °C overnight. Then, the medium was aspirated, 500 μL of 10 ug/mL Cu-Cy-I nanoparticles were added, and the cells were continuously cultured for 6 h followed by 2 Gy of X-ray irradiation at a dose rate of 1.212 Gy/min with the RS 2000 X-ray irradiator. Thirty minutes later, the cells were fixed with 4% paraformaldehyde, washed 3 times with PBS, permeabilized with 0.1% Triton X-100 for 10 min, and subsequently washed 3 times with PBS. Then, the cells were blocked with 2% bovine serum albumin for approximately 60 min, followed by 3 washes with PBS, and incubated overnight in a humidified chamber with a mouse monoclonal anti-phospho-Histone H_2_AX antibody diluted at 1:100. Subsequently, a 1:500 diluted Alexa Fluor 647-conjugated goat anti-mouse secondary antibody was added and the cells were incubated in darkness for 1 h. After mounting with a DAPI-containing anti-fade mounting medium, the cells were observed with the confocal laser scanning microscope. Fluorescent microscopic images were acquired at Ex 305 nm/Em 461 nm for DAPI-stained nuclei and Ex 635 nm/Em 668 nm for γ-H_2_AX foci inside the cell.

### 2.8. In Vitro Cytotoxicity Analysis

The 4T1, ID8, OVCAR3, and A2780 cells were employed to assess the in vitro cytotoxic effects of Cu-Cy-I nanoparticles alone and in combination with X-ray irradiation. The in vitro cultured cells were digested with a 0.25% trypsin–EDTA solution, then suspended in DMEM medium and seeded in a 96-well plate at a density of 6000 cells per well. After overnight culturing at 37 °C to allow cell attachment, the DMEM medium in the wells was aspirated and freshly prepared DMEM media containing various concentrations of Cu-Cy-I nanoparticles (0, 3.13, 6.25, 12.50, 25.00, 50.00, and 100 ug/mL) were added into the wells. The X-ray irradiation group was also exposed to 4 Gy of X-ray irradiation 4 h after the addition of the Cu-Cy-I nanoparticles. Both the X-ray-irradiated and not-irradiated cells were cultured for 24 h and then the cell viability was assessed by CCK-8 assay, where 10 µL of CCK-8 solution and 90 μL of DMEM medium were added to each well and the visible absorbance at 450 nm of the well was measured after 2 h of incubation. The cell viability measured by CCK-8 assay was calculated according to the following formula: cell viability = [(As − Ab)/(Ac − Ab)] × 100%, where As, Ac, and Ab refer to the absorbance values of the experimental group, the cells without adding the nanoparticles or IR as the control group, and the culture medium as the blank group, respectively.

### 2.9. In Vivo Antitumor Assessment

An orthotopic mouse breast cancer model was setup with 4T1 cells and 6–8 week-old Balb/c mice. A suspension of 1 × 10^6^ 4T1 cells in 100 µL PBS was orthotopically inoculated into the fourth mammary gland on the left side of the mice. While the tumor volume approached 200–300 mm^3^, the mice were randomly divided into 5 groups (6 mice for each group). The control group was intratumorally injected with 100 μL of PBS. The four treatment groups included the Cu-Cy-I nanoparticles group, X-ray group, Cu-Cy-I nanoparticles and X-ray group, and [^131^I]Cu-Cy-I nanoparticles group. The Cu-Cy-I nanoparticles suspended in PBS (0.8 mg/mL) were injected slowly into each tumor with the needle inserted at about 5 mm depth from the tumor surface at a Cu-Cy-I dose of 40 µg/100 μL tumor volume. The X-ray irradiation with the RS 2000 X-ray irradiator totaled 20 Gy to the tumor at an irradiation dose rate of 4.5 Gy/min, 5 Gy every 3 days. The first X-ray irradiation was performed 30 min post-nanoparticle injection. [^131^I]Cu-Cy-I nanoparticles were intratumorally injected at doses of 40ug Cu-Cy-I and 50 µCi of ^131^I /100 μL tumor volume. The body weights and tumor sizes were measured every two days. Tumor volumes were calculated using the following formula: volume = (width^2^ × length)/2. The survival periods were recorded until all the mice lived to the experimental endpoint, with the criteria being animal death or the tumor size reaching a volume of 1500 mm^3^ or length of 15 mm, and the survival rate of each group was calculated. The average survival times after each treatment were determined using Kaplan–Meier analysis with Graphpad Prism 9.5 software.

Tumor samples were dissected at the end point of the animal experiment and cut into 2 parts using a sterile blade from a middle position roughly along the direction of the needle entry of the intratumoral injection of the nanoparticles. White light images and the visible fluorescent images under UV lamp excitation of the dissected tumors were acquired with a digital camera, respectively.

### 2.10. Statistical Analysis

All results were presented as the mean ± standard deviation (SD). Statistical analysis was conducted using GraphPad Prism 9.5 software. Differences between groups were analyzed using *t*-tests and the levels of statistical significance of difference were set at probabilities of * *p* < 0.05, ** *p* < 0.01, *** *p* < 0.001, and **** *p* < 0.0001.

## 3. Results

### 3.1. Synthesis and Characterization of Cu-Cy-I Nanoparticles

**Figure 1:**
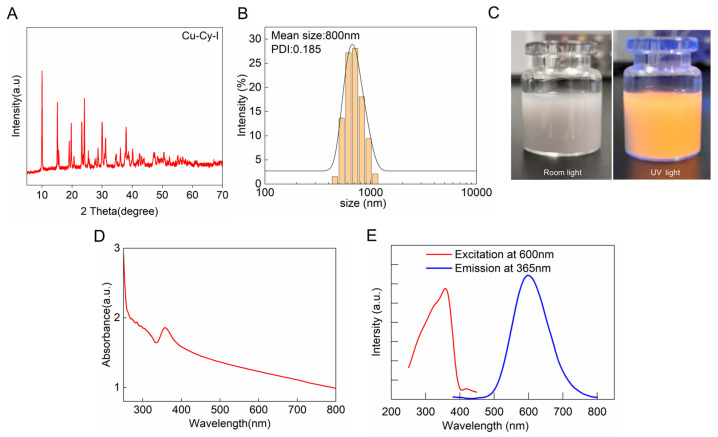
Characterization and optical properties of Cu-Cy-I nanoparticles. (**A**) XRD spectra of Cu-Cy-I. (**B**) Particle size and PDI measured by DLS. (**C**) Photographs of Cu-Cy-I under white light and 365 nm UV light. (**D**) UV–visible absorption spectrum. (**E**) Fluorescence excitation spectrum by emission light of 600 nm wavelength (red line) and emission spectrum by excitation at 365 nm UV light (blue line).

The Cu-Cy-I nanoparticles were synthesized with reference to a previous method, with some modifications [27]. We used copper acetate as the copper source instead of copper chloride to reduce the competition of chloride with iodide during the formation of the nanoparticles. Moreover, the pH buffer capacity of CuAc_2_ made the adjustment of the reaction system at pH 7.0 easier. PVA was added to the reaction to improve the dispersed formation of the nanoparticles. The synthesized product in the reaction mixture system exhibited yellow fluorescence upon UV irradiation. The XRD spectrum of the purified product indicated the strongest peak of Cu-Cy-I at 2 theta = 10.07° (Figure 1A), which was consistent with the literature [27], confirming the successful preparation of Cu-Cy-I nanoparticles. The recovery of iodide ions in the Cu-Cy-I nanoparticles was 15%, much higher than the value of 3.7% estimated from the literature.

The hydrodynamic particle diameters of Cu-Cy-I nanoparticles suspended in water measured by DLS were approximately 800 nm, having a narrow distribution with a PDI < 0.2 (Figure 1B). The Cu-Cy-I nanoparticles emitted strong yellow luminescence under 365 nm UV light irradiation (Figure 1C). The UV–visible spectrum of the nanoparticles had an absorption band maximized at about 360 nm, which could be attributed to the metal-to-ligand charge transfer (MLCT) or metal-centered (MC) emission [30]. The fluorescent emission spectrum under 365 nm UV light excitation showed a strong emission peak at 600 nm (Figure 1D,E), a little bit blue-shifted from previously reported results [27], which may have been partially affected by the PVA coating of the nanoparticles.

### 3.2. Synthesis of [^131^I]Cu-Cy-I Nanoparticles and the Radiolabeling Stability

**Figure 2:**
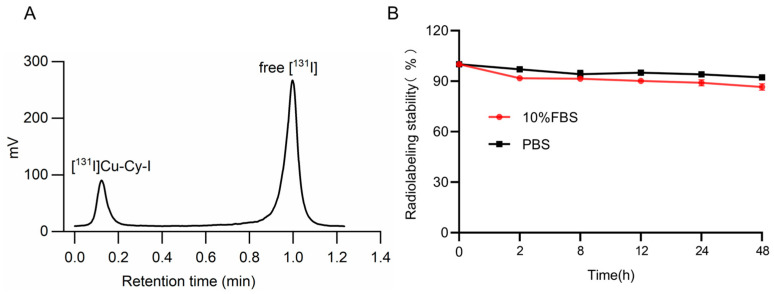
(**A**) Radio-TLC results of the radiolabeling mixture, purified [^131^I]Cu-Cy-I nanoparticles and free [^131^I]I^−^. (**B**) Radiolabeling stability of [^131^I]Cu-Cy-I in PBS and 10% FBS.

The ^131^I-labeled Cu-Cy-I nanoparticles were synthesized by adding [^131^I]NaI stock solution to the KI solution and following a similar procedure for the synthesis of unlabeled Cu-Cy-I nanoparticles. During the synthesis, the reaction system was examined by UV light irradiation to ensure the formation of the fluorescent nanoparticles. Free [^131^I]I^−^ migrated together with the solvent front, while [^131^I]Cu-Cy-I stayed at the loading spot during the solvent expansion of radio-TLC. The radiolabeling efficiency measured by radio-TLC was 23.9% (Figure 2A). The radiochemical yield of the collected product was about 21.8%.

The percentages of ^131^I retained in [^131^I]Cu-Cy-I nanoparticles were about 92% and 86%, respectively, in either PBS or 10% FBS within 48 h (Figure 2B), indicating that the ^131^I labeling of the nanoparticles was stable, only having a slow and minor dissociation with time.

### 3.3. In Vitro ROS Generation in Tumor Cells Caused by Cu-Cy-I Nanoparticles and X-Ray Irradiation

**Figure 3:**
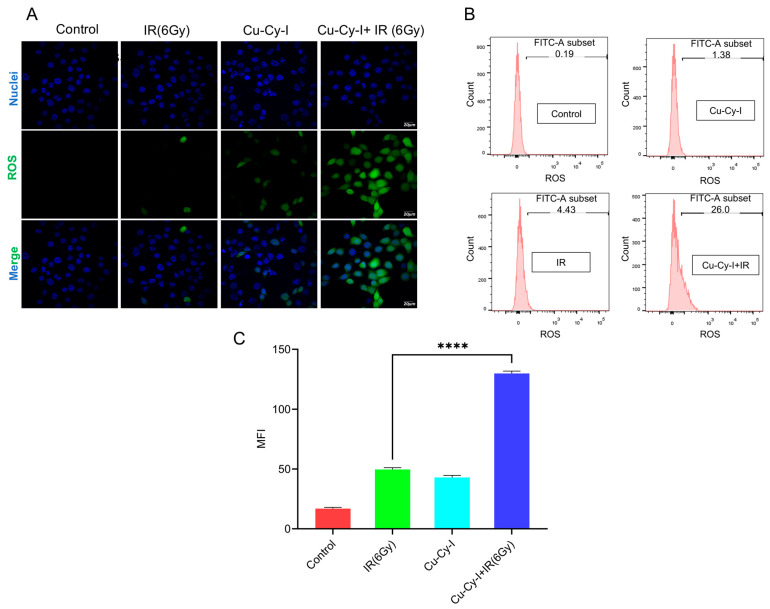
The production of ROS in 4T1 cells after different treatments detected with DCFH-DA probe by (**A**) confocal fluorescence microscope. (**B**) Flow cytometer. (**C**) Fluorescence quantification data of DCF intensity by flow cytometer (MFI, mean fluorescence intensity). **** *p* < 0.0001.

The production of ROS by tumor cells in the presence of Cu-Cy-I nanoparticles upon X-ray irradiation was investigated using the DCFH-DA probe, which could cross cell membranes and be oxidized by ROS to produce the fluorescent DCF [31]. The confocal microscopic images showed that the tumor cells irradiated by X-ray or cultured with Cu-Cy-I nanoparticles produced a weak DCF fluorescent signal; however, the tumor cells in the presence of Cu-Cy-I nanoparticles irradiated by X-ray generated a much stronger DCF fluorescent signal (Figure 3A), indicating the Cu-Cy-I nanoparticles were efficiently activated by X-ray irradiation. Such a result was also confirmed by flow cytometry assay (Figure 3B), indicating Cu-Cy-I nanoparticles are an effective ROS enhancer under X-ray irradiation.

### 3.4. Formation of γH_2_AX Foci in Tumor Cells Caused by Cu-Cy-I Nanoparticles and X-Ray Irradiation

**Figure 4:**
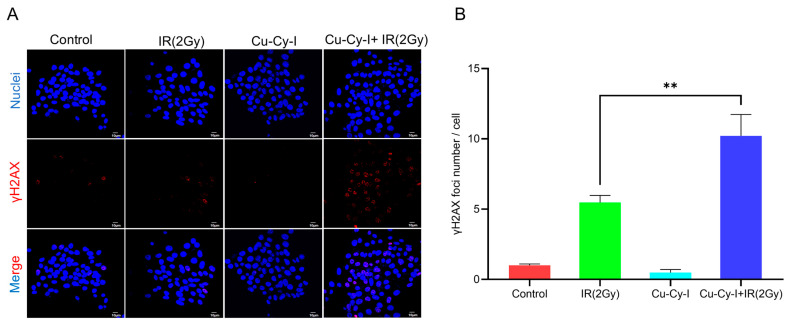
Cu-Cy-I nanoparticles enhanced radiation-induced DNA damage in 4T1 cells receiving Cu-Cy-I and IR (2Gy). (**A**) Representative immunofluorescence images of γH_2_AX foci. (**B**) Quantification data of γH_2_AX foci. ** *p* < 0.01.

γH_2_AX is a sensitive biomarker of oxidative DNA damage caused by ROS. The confocal fluorescent microscopic images showed that the formation of γH_2_AX foci was almost neglectable inside the 4T1 tumor cells incubated with Cu-Cy-I nanoparticles and was not increased much inside the cells exposed to 2 Gy of X-ray irradiation. Comparably, the formation of γH_2_AX foci was significantly elevated in the cells under the combined treatment with Cu-Cy-I nanoparticles and X-ray irradiation (Figure 4).

### 3.5. In Vitro Tumor Cell Toxicity of Cu-Cy-I Nanoparticles with X-Ray Irradiation

**Figure 5:**
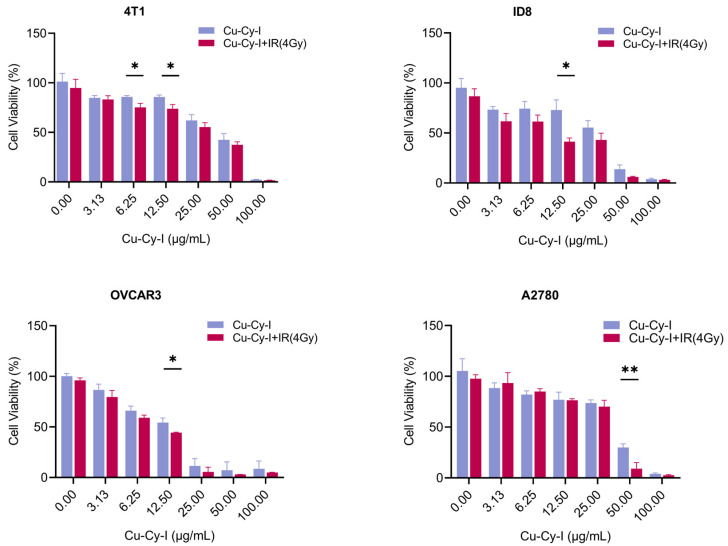
The cytotoxicity of Cu-Cy-I nanoparticles and X-ray irradiation to tumor cells in vitro as measured by CCK-8 assay. * *p* < 0.05, ** *p* < 0.01.

The cytotoxicity of Cu-Cy-I nanoparticles to tumor cells was evaluated with CCK-8 assay. The cell viability decreased with increasing amounts of Cu-Cy-I nanoparticles in the medium for all the cell lines (Figure 5). The Cu-Cy-I nanoparticles showed weak dark cytotoxicity to all of the four cell lines. The 4 Gy of X-ray irradiation alone did not significantly affect the tumor cell viability, as observed at 24 h post-irradiation. However, the X-ray irradiation of the tumor cells incubated with Cu-Cy-I nanoparticles resulted in higher cytotoxicity as compared to the non-irradiated cells in the presence of the same concentration of the nanoparticles (Figure 5), indicating the nanoparticles enhanced the sensitivity of tumor cells to X-ray irradiation.

### 3.6. In Vivo Antitumor Effect

**Figure 6:**
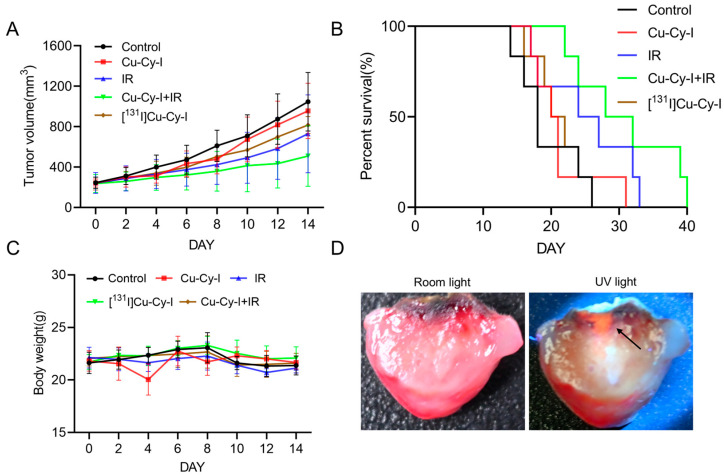
In vivo antitumor effect on the orthotopic 4T1 breast cancer cell-xenografted mice. (**A**) Tumor sizes, (**B**) survival curve, (**C**) body weight, and (**D**) distribution of Cu-Cy-I nanoparticles within the tumor (black arrow).

The encouraging in vitro results inspired us to further investigate the in vivo antitumor potential of Cu-Cy-I and [^131^I]Cu-Cy-I nanoparticles. The radiosensitivity effects of Cu-Cy-I nanoparticles companying either the X-ray radiotherapy or radionuclide therapy were assessed. For the post-intratumoral administration of Cu-Cy-I at a drug dose of 40 µg/100 μL of tumor volume to the Balb/c mice, the mean tumor volumes on day 4 to 14 were a little bit smaller than the control groups, but had no statistical difference during the observation period, indicating the minor tumor growth-inhibition effect of Cu-Cy-I nanoparticles alone. The total of 20 Gy of X-ray radiotherapy significantly delayed the tumor growth and the combination of Cu-Cy-I nanoparticles and X-ray radiotherapy caused the strongest tumor growth-inhibition effect among all the groups, reflecting the radiosensitivity efficiency of Cu-Cy-I nanoparticles inside tumor tissue for enhanced X-ray radiotherapy. However, the intratumoral administration of ^131^I-labeled [^131^I]Cu-Cy-I at same drug dose as the Cu-Cy-I group and additive 50 µCi ^131^I/100 μL of tumor volume seemed to produce a tumor growth-delaying effect stronger than the Cu-Cy-I nanoparticles group, but without statistical significance (Figure 6A).

Survival analyses of mice of the five groups resulted in a sequence of the median survival time: Cu-Cy-I nanoparticles and X-ray group (30 days) > X-ray group (25.5 days) > [^131^I]Cu-Cy-I nanoparticles group (21 days) > Cu-Cy-I nanoparticles group (20.5 days) > control group (18 days) (Figure 6B), indicating that the Cu-Cy-I nanoparticles and X-ray combined therapy could prolong the survival of 4T1 tumor-xenografted mice most efficiently in the experimental groups. The body weights of all the groups did not change much during the 14 d observation period (Figure 6C), indicating that the treatments with the nanoparticles had no obvious systemic toxicity to the mice.

As the radionuclide therapy with [^131^I]Cu-Cy-I nanoparticles did not markedly delay the tumor growth or extend the mouse median survival time compared to both the control group and Cu-Cy-I nanoparticles group, we further observed the distribution of the fluorescent Cu-Cy-I nanoparticles inside the cut pieces of tumor dissected at the experimental endpoint. The administrated nanoparticles were observed to be irregularly deposited in a limited area around the injection site and did not diffuse to the tumor rim (Figure 6D).

## 4. Discussion

We successfully synthesized fluorescent Cu-Cy-I nanoparticles using copper acetate as the copper source, with a significant improvement of the recovery of iodide ion in the nanoparticles, and labeled the Cu-Cy-I nanoparticles with ^131^I with a radiochemical yield of about 22%. The [^131^I]Cu-Cy-I nanoparticles were stable in either PBS or 10% FBS media, assuring that they are appropriate candidates for the investigation of their radiosensitization potentials for tumor radiotherapy.

The Cu-Cy-I nanoparticles were efficiently activated by 6 Gy of X-ray irradiation to enhance the ROS production of 4T1 tumor cells in vitro, as detected with a DCFH-DA probe. Moreover, the combined treatment of 4T1 cells with Cu-Cy-I nanoparticles and 2 Gy of X-ray irradiation also resulted in the elevated formation of intracellular γH_2_AX foci, which reflected the degree of ROS-induced oxidative DNA damage. These in vitro experimental results suggest that Cu-Cy-I nanoparticles are potential photosensitizers for improving the therapeutic efficiency of X-ray radiotherapy.

Increased in vitro cytotoxicity was observed in the 4T1, ID8, OVCAR3, and A2780 tumor cells treated with some concentrations of Cu-Cy-I nanoparticles and 4 Gy of X-ray irradiation. This result may be correlated with the elevated ROS level and DNA damage marked by increased γH_2_AX foci in the tumor cells under the combined treatment of Cu-Cy-I nanoparticles and X-ray irradiation [32].

Intratumoral radiotherapy with therapeutic radionuclide-carrying nanoparticles has been developed to efficiently treat locally refractory tumors, taking advantage of the cross-fire effect of radionuclides and the diffusion and retention properties of nanoparticles [33,34,35]. We investigated the tumor growth-inhibition effects of intratumorally administrated Cu-Cy-I nanoparticles as an X-ray-responsive phostosensitizer and therapeutic radionuclide ^131^I-labeled [^131^I]Cu-Cy-I nanoparticles for the potential combination of radiotherapy and X-ray-induced or radionuclide-induced photodynamic therapy.

The in vivo treatment results with orthotopic 4T1 tumor-xenografted mice indicated that intratumorally administrated Cu-Cy-I nanoparticles had a neglectable therapeutic effect, but the combination of the nanoparticles with 20 Gy of X-ray radiotherapy improved significantly the delay of tumor growth, indicating Cu-Cy-I nanoparticles are an efficient radiosensitive nanomaterial for enhanced X-ray radiotherapy. The improved tumor growth-inhibition effect and low systemic toxicity of the Cu-Cy-I and X-ray combination group support the use of Cu-Cy-I nanoparticles as a radiosensitizer in combination with X-ray radiotherapy.

However, the intratumoral administration of [^131^I]Cu-Cy-I nanoparticles did not result in a statistically significant tumor growth delay compared to Cu-Cy-I. One possible reason, other than the radiosensitization characterization of [^131^I]Cu-Cy-I, is that the ^131^I radioactivity of the administrated nanoparticles was at a low level. However, other factors effecting the therapeutic effect should be considered. As the microdistribution of the radionuclide inside the tumor constrains the effective radiation dose and therapeutic efficiency of intratumorally administrated radionuclides [33,34,35], we observed the dissected tumors at the experimental endpoint and found the accumulation of intratumorally administrated [^131^I]Cu-Cy-I nanoparticles around the injection site, which indicates the limited diffusion of the nanoparticles inside the tumors. Since the average β range of ^131^I in soft tissue is only 0.91 mm [36], the radiation dose decreased rapidly with increasing distance from the [^131^I]Cu-Cy-I nanoparticles deposited at the injection site; thus, the growth of the tumor rim not close to the deposition zone of the nanoparticles could not be efficiently hurt by the ^131^I radionuclide therapy and expected ^131^I-induced Cu-Cy-I photodynamic therapy.

Though the antitumor efficacy of ^131^I-induced Cu-Cy-I radiotherapy and photodynamic therapy was not verified by the current study, this modality has the benefits of simple treatment and potentially low side effects compared to exterior radiotherapy. The size of the nanoparticles is a crucial feature effecting the pharmacokinetics and biodistribution after either intravenous injection or intratumoral injection. Nanoparticles of around 100 nm or sub-100 nm can penetrate and accumulate inside tumor tissue more efficiently than bigger sizes of nanoparticles [37,38]. The relatively big sizes (about 800 nm) of the Cu-Cy-I nanoparticles made them hardly diffuse inside the tumor tissue and may also limit uptake of the nanoparticles by tumor cells, thus leading to an insignificant therapeutic effect. Smaller sizes of the nanoparticles are beneficial to approach the uniform intratumoral ^131^I distribution and optimize the radiation dosimetry inside the tumor. Moreover, ROS are more likely to injure cells intracellularly compared to extracellularly and the subcellular localization of a photosensitizer within cells has been suggested to be a primary determinant of the site of initial photodynamic damage in PDT [39]. Thus, the use of smaller sizes of nanoparticles, enabling their efficient uptake by tumor cells, is expected to improve the intracellular radionuclide-induced photodynamic damage.

## 5. Conclusions

Cu-Cy-I nanoparticles were synthesized conveniently with a modified route which was also used for the successful preparation of [^131^I]Cu-Cy-I nanoparticles with an acceptable radiolabeling yield. In vitro and in vivo experiments indicated that Cu-Cy-I nanoparticles have good potential as a new type of radiosensitizer to enhance the X-ray radiotherapy efficacy. The [^131^I]Cu-Cy-I nanoparticles had good radiolabeling stability. However, the [^131^I]Cu-Cy-I nanoparticles deposited mostly around the injection site inside tumors and the efficacy of local therapy with [^131^I]Cu-Cy-I nanoparticles at a low ^131^I dose was not verified by the in vivo experiment with a mouse model. As the particle size is a critical feature, affecting the diffusion and microdistribution of nanoparticles inside tumors, the synthesis of smaller sizes of nanoparticles, ideally around 100 nm or even smaller, is necessary for the further investigation of the potential of combined radiotherapy and radionuclide-induced PDT with [^131^I]Cu-Cy-I nanoparticles.

## Data Availability

The original contributions presented in this study are included in the article. Further inquiries can be directed to the corresponding author.
